# Support Vector Machine-Based Prediction Model for Healthcare Workforce Transition Success Under Decentralization

**DOI:** 10.12688/f1000research.160378.2

**Published:** 2025-09-24

**Authors:** Atiya Sarakshetrin, Chinakorn Sujimongkol, Daravan Rongmuang, Rungnapa Chantra, Suchada Nimwatanakul

**Affiliations:** 1 Faculty of Nursing, Praboromarajchanok Institute, Nonthaburi, Thailand; 2 Faculty of Public Health and Allied Health Sciences, Praboromarajchanok Institute, Nonthaburi, Thailand

**Keywords:** Health Personnel, Decentralization, Support Vector Machine, Predictive Model

## Abstract

**Objective:**

To develop and validate predictive models for healthcare workforce transition success under decentralization using Support Vector Machine (SVM) analysis and to identify key determinants across organizational support domains.

**Methods:**

A cross-sectional study was conducted among 430 healthcare personnel transferred from Ministry of Public Health facilities to Provincial Administrative Organizations in Thailand (2023–2024). Thirty-seven predictors, including demographics, benefits, and welfare domains, were analyzed. Four kernel functions were compared using 10-fold cross-validation, and feature importance was assessed. Class imbalance was addressed with the Synthetic Minority Oversampling Technique (SMOTE).

**Results:**

The linear kernel achieved superior cross-validated performance (accuracy: 69 ± 4%, sensitivity: 46 ± 5%, specificity: 82 ± 4%, AUC: 0.64). SMOTE improved sensitivity to 54 ± 5% while maintaining specificity at 79 ± 5%. Five stable predictors were identified across validation folds: competitive compensation (0.427), career development opportunities (0.358), fair promotion processes (0.336), hazardous work compensation (0.285), and educational leave opportunities (0.252). Comparative analysis showed that SVM outperformed logistic regression (66% accuracy), random forest (66%), and gradient boosting (65%).

**Conclusions:**

This study represents the first application of machine learning techniques to predict healthcare personnel transition success in decentralization contexts. The SVM model effectively identified critical factors influencing workforce transitions, emphasizing the importance of balanced organizational support mechanisms. These findings provide evidence-based guidance for healthcare administrators implementing decentralization policies, offering generalizable insights for workforce management during health system reforms.

## Introduction

Healthcare decentralization has emerged as a significant global trend in health system reform, with various models implemented across both developed and developing countries. A prominent approach involves transferring administrative authority and resource management from central ministries to local administrative organizations. This transition, existed across various countries ( Dwicaksono & Fox, 2018; Jiménez-Rubio, 2023; Muñoz et al., 2017), aims to enhance healthcare delivery through local governance and community-responsive management ( Dougherty et al., 2022; Jiménez-Rubio & García-Gómez, 2017). A critical element of successful decentralization is human resource management, particularly the transfer of healthcare personnel from centralized to local administrative control. The transition of healthcare workers represents one of the most challenging aspects of decentralization, as it directly impacts service delivery quality and health system performance. Understanding healthcare workers’ perspectives and experiences during this transition is crucial, as their successful adaptation to decentralized systems significantly influences the overall effectiveness of health sector reform. Healthcare organizations face complex challenges in managing professional transitions during decentralization, especially regarding workforce welfare and career development opportunities. These challenges are often compounded by limited planning instruments, resource constraints, and inadequate guidelines for professional development. A critical aspect of successful healthcare decentralization lies in effective workforce management and transition planning ( Sohag & Miankhel, 2013). Healthcare professionals’ adaptation to decentralized systems significantly impacts service delivery quality and organizational sustainability. However, the complexity of workforce transitions involves multiple interrelated factors affecting benefits, welfare, and career advancement domains. Understanding healthcare workers’ perspectives and experiences during this transition is crucial, as their successful adaptation to decentralized systems significantly influences the overall effectiveness of health sector reform. Traditional analytical approaches often struggle to capture these intricate relationships, particularly given the diversity and multidimensional nature of impact factors in the decentralization process. Furthermore, the lack of proper data for evidence-based decision-making at local levels presents additional challenges in predicting and managing workforce transitions effectively ( Sarti, 2023).

Recent advances in machine learning, particularly Support Vector Machines (SVMs), offer promising analytical approaches for understanding complex healthcare workforce transitions. This study applies SVM methodology to identify key determinants of successful personnel transitions during healthcare decentralization, focusing on factors affecting workforce satisfaction and retention. SVMs have shown remarkable success in various healthcare applications, from disease diagnosis to outcome prediction ( Guido et al., 2024). The effectiveness of SVM in handling multiple variables and achieving high prediction accuracy makes it particularly suitable for analyzing complex healthcare management scenarios ( Bagul et al., 2024). This capability is especially relevant in workforce management predictions, where multiple factors influence outcomes. The robust predictive capabilities of SVM in handling multidimensional healthcare data ( Gund et al., 2023) suggested its potential value in analyzing workforce transitions, where multiple factors influenced professional success.

To date, SVM modeling has not been applied to predict healthcare personnel transition success in decentralized health systems, either in Thailand or internationally. While traditional analytical methods have been used to study healthcare decentralization outcomes, the application of machine learning approaches, particularly SVM, remains unexplored in this context. This study investigated the use of an SVM-based classification model to determine predictors of successful workforce transitions across benefits, welfare, and career advancement domains in Thailand’s decentralized healthcare system, with the ultimate goal of providing evidence-based insights for optimizing workforce management strategies in decentralized healthcare systems. The study addressed two key objectives:

1. Development of validated predictive models for professional transition success by analyzing multiple domains (benefits, welfare, and career advancement)
2. Identification of key factors influencing workforce adaptation through SVM classification, enabling early detection of potential challenges and success factors

## Methods

### Study design and population

This was a cross-sectional study that focused on quantitative analysis, complementing a previously published qualitative investigation from our larger project on Fringe Benefits, Welfare, and Career Paths of Personnel in Health Promotion Hospitals under Provincial Administrative Organization (published elsewhere), conducted between March to October 2023. The study aimed to develop predictive models using Support Vector Machine (SVM) analysis to identify factors influencing workforce transition success during Thailand’s healthcare decentralization process.

### Sampling strategy

Eight provinces were strategically selected from Thailand’s 77 provinces, representing three levels of healthcare decentralization implementation: low (less than 50% of districts within the province had transferred healthcare facilities to local organizations), moderate (50-99% of districts had completed transfers), and full implementation (all districts within the province had completed transfers to local organizations). These levels were represented by four, two, and two provinces respectively. Sample size was calculated using population proportion estimation (95% confidence interval, ±5% precision) with 15% adjustment for non-response, yielding 430 participants.

### Survey instrument

A validated structured questionnaire was developed comprising two main sections. The first section collected demographic and organizational characteristics, including participants’ age, marital status, education level, monthly income, work experience, current position, facility staff headcount (pre- and post-decentralization), number of registered nurses, and facility capacity classification. The second section assessed satisfaction across benefits (19 items) and welfare (11 items) domains, evaluating aspects such as compensation, career advancement, and professional development opportunities using a 5-point Likert scale (1 = very dissatisfied to 5 = very satisfied). The instrument demonstrated strong psychometric properties, with an Item-Objective Congruence Index of 0.8-1.0 for content validity and a Cronbach’s alpha coefficient of 0.96 from pilot testing with 30 non-study healthcare facilities.

### Ethical considerations

The study protocol was approved by the Ethics Committee for Human Research of PCKCN (approval number: REC No. 13/2566, dated March 23, 2023). All participants provided informed consent prior to data collection.

### Support vector machine model development

The study used SVM analysis in R statistical software (version 4.0.2, e1071 package) to develop a predictive model for workforce transition success. Model development included data preprocessing through standardization of 37 predictor variables spanning demographic factors, benefits, and welfare domains. Four kernel functions (linear, radial basis function, polynomial, and sigmoid) were evaluated to determine optimal model performance.

### Outcome variable definition

The binary outcome “successful transition” was operationalized using a composite satisfaction score approach;

• Successful transition: Mean satisfaction score ≥ 3.5 across all benefits and welfare domain items
• Unsuccessful transition: Mean satisfaction score < 3.5

The threshold of 3.5 was substantiated by a preliminary ROC analysis (not presented here), which demonstrated that this cutoff offered an appropriate balance between sensitivity and specificity for classifying transition success. Notably, 3.5 also represents the midpoint between ‘neutral’ and ‘satisfied’ on the Likert scale, rendering it both a statistically and conceptually significant threshold. The final distribution of outcomes was as follows: successful transition (n=195, 45.3%) and unsuccessful transition (n=235, 54.7%).

The linear kernel function was selected based on comparative performance metrics:

$$f\left(x\right)=\mathrm{sign}\left(\sum_{i=1}^{n}\;\;\alpha_{i}y_{i}\;K\left(x_{i},x\right)+b\right)$$

where *f* ( *x*) represents the decision function classifying workforce transition success (success/unsuccessful), *K*( *x*_*i*_, *x*) is the linear kernel function, *α*_*i*_ are the Lagrange multipliers, *y*_*i*_ are the class labels, and b is the bias term ( Cortes & Vapnik, 1995). Feature importance analysis was conducted using the weight vector of the linear SVM model to identify key predictors of successful transitions.

Model performance was assessed using three metrics: accuracy for measuring overall correct classification rate, sensitivity for assessing true positive rate of successful transitions, and specificity for evaluating true negative rate of unsuccessful transitions. Feature importance analysis was subsequently performed using the weight vectors of the selected kernel model to identify key predictors of successful transitions.

### Class imbalance assessment and handling

To address the identified moderate class imbalance (successful transitions: unsuccessful), we implemented the Synthetic Minority Oversampling Technique (SMOTE) during the training phases. The SMOTE was employed to balance the dataset by generating synthetic examples of the minority class. This process, which involves interpolating between existing successful transition cases, was conducted to enhance the model’s sensitivity for successful outcomes.

### Comparative model assessment

To validate the SVM selection, we compared the cross-validated performance with that of alternative machine learning algorithms.

• Logistic Regression with L2 regularization
• Random Forest (n_estimators = 100)
• Gradient Boosting Classifier (n_estimators = 100)

All models underwent identical preprocessing and cross-validation procedures to ensure fair comparison.

### Performance evaluation metrics

Model performance was assessed using standard classification metrics: accuracy for the overall correct classification rate, sensitivity for the true positive rate of successful transitions, specificity for the true negative rate of unsuccessful transitions, F1-score for balanced precision-recall performance, and Area Under the Curve (AUC) for overall discriminative ability.

## Results

### Demographic characteristics

Of the 430 healthcare personnel studied, the majority were female (78.60%, n=338) with a bimodal age distribution peaking at 25-35 years (34.88%, n=150) and over 45 years (33.49%, n=144). More than half were married (56.51%, n=243), and nearly three-quarters held bachelor’s degrees (71.16%, n=306). Professional experience was substantial, with approximately one-third having over 20 years of service (32.79%, n=141). The workforce composition primarily comprised public health officers (23.02%, n=99) and registered nurses (10.47%, n=45), with most personnel (58.14%, n=250) serving in medium-sized sub-district health promoting hospitals.

### Predictive factors for workforce transition success

Cross-validation analysis of feature importance revealed stable rankings for the top predictors, with a coefficient of variation <0.15 for the five highest-weighted features, confirming the robustness of the identified determinants. The target variable was defined as successful transition based on improvements in personnel satisfaction across benefits, welfare, and career advancement domains after transferring to work under the Provincial Administrative Organization.

Feature importance analysis of the 37 predictor variables (10 demographic/organizational, 16 benefits, and 11 welfare variables) using the linear kernel SVM model revealed the relative importance of predictors as shown in Table 1.

**Table 1.** SVM-identified predictors of healthcare workforce transition success.

Rank	Feature	Description	Feature weight *	CV Range
1	Benefits5	Competitive compensation and benefits	0.427	0.398-0.456
2	Welfare30	Career development opportunities	0.358	0.334-0.382
3	Benefits4	Fair and transparent promotion processes	0.336	0.312-0.360
4	Benefits15	Fair compensation for hazardous work	0.285	0.265-0.305
5	Welfare20	Educational leave opportunities	0.252	0.235-0.269
6	Welfare21	Professional development opportunities	0.239	0.221-0.257
7	Education ^†^	Educational level	0.236	0.218-0.254
8	Benefits10	Flexible work arrangements	0.197	0.182-0.212
9	Benefits16	Recognition and rewards for performance and contributions	0.196	0.181-0.211
10	Welfare26	Employee wellness programs	0.190	0.175-0.205

^*^ Feature weights derived from linear SVM coefficient magnitudes, normalized to [0,1] scale, indicating relative predictive importance. CV Range = Cross-validation range across 10 folds.

^†^ Education is a demographic variable, while others are satisfaction assessment items.

Analysis of feature weights derived from the SVM model identified ten key predictors of workforce transition success ( Table 1), with coefficients ranging from 0.427 to 0.190. Financial considerations demonstrated the strongest predictive power, with competitive compensation and benefits (Benefits5, coefficient=0.427) emerging as the primary determinant. Career development opportunities (Welfare30, coefficient=0.358) ranked as the second most influential predictor, suggesting that successful transitions are driven by both immediate financial incentives and long-term professional growth prospects. Among demographic characteristics, educational qualification (coefficient=0.236) emerged as a significant predictor, highlighting the role of individual capacity in transition outcomes. The hierarchical distribution of feature weights provides evidence-based guidance for prioritizing workforce management interventions in decentralized healthcare systems.

### Kernel performance summary

Based on the five highest-ranked predictors (feature weights 0.427-0.252) identified through SVM analysis, we evaluated classification performance using four different kernel functions (linear, RBF, polynomial, and sigmoid). These key predictors encompassed competitive compensation (Benefits5), career development opportunities (Welfare30), promotion processes (Benefits4), hazardous work compensation (Benefits15), and educational opportunities (Welfare20). Table 2 presents the comparative performance metrics, where the linear kernel demonstrated superior cross-validated performance with optimal accuracy and balanced sensitivity-specificity trade-off. While the RBF kernel showed comparable results, the linear kernel’s combination of performance and simplicity made it the preferred choice for our workforce transition prediction model.

**Table 2.** Performance comparison of SVM kernel functions.

Kernel type	Performance metrics of different SVM kernels (%)			AUC
	CV * Accuracy	CV Sensitivity	CV Specificity	
Linear	68.5 ± 3.8	46.1 ± 5.2	82.4 ± 4.1	0.642
Radial	66.8 ± 4.1	44.2 ± 5.8	80.9 ± 4.3	0.625
Polynomial	64.2 ± 4.6	16.8 ± 6.2	95.1 ± 2.9	0.559
Sigmoid	54.8 ± 5.2	39.3 ± 6.1	64.2 ± 5.8	0.518

* CV = Cross-validation (10-fold); values show mean ± standard deviation across folds. The linear kernel demonstrated an optimal balance between accuracy and interpretability for workforce transition prediction.

### Cross-validation performance and model comparison

Cross-validation analysis demonstrated stable model performance across the different data partitions ( Table 3). The linear SVM achieved a cross-validated accuracy of 68.5±3.8%, representing approximately 3% degradation from single-fold performance, which is typical for datasets of this size and complexity.

**Table 3.** Cross-validation performance comparison.

Model	CV * Accuracy (%)	CV Sensitivity (%)	CV Specificity (%)	AUC ^†^
Linear SVM	68.5 ± 3.8	46.1 ± 5.2	82.4 ± 4.1	0.642
SVM + SMOTE	66.8 ± 4.2	54.4 ± 4.8	78.9 ± 4.5	0.666
Logistic Regression	66.2 ± 4.0	43.8 ± 5.0	81.1 ± 4.3	0.625
Random Forest	65.8 ± 4.5	48.9 ± 5.8	77.2 ± 4.8	0.631
Gradient Boosting	65.1 ± 4.3	47.2 ± 5.5	78.5 ± 4.2	0.628

* CV = Cross-validation.

^†^ AUC = Area Under Curve.

### Class imbalance analysis and SMOTE implementation

The outcome distribution revealed a moderate imbalance, with successful transitions comprising 45.3% (n=195) and unsuccessful transitions 54.7% (n=235) of the cases. Implementation of SMOTE during training phase improved sensitivity from 46 % to 54 % while maintaining reasonable specificity of 78%. This improvement in balanced performance provides more clinically relevant metrics for workforce management decision-making ( Table 4).

**Table 4.** Confusion matrix for optimal SVM model (SMOTE-enhanced).

Actual	Predicted Unsuccessful	Predicted Successful	Total
Unsuccessful	185	50	235
Successful	89	106	195
**Total**	274	156	430

The performance of the SMOTE-enhanced optimal SVM model was evaluated using the confusion matrix presented in Table 4. The model’s accuracy was 67.0%, calculated as (TP+TN)/Total. Further metrics were derived, including a sensitivity (recall) of around 54% (TP/(TP+FN)), a specificity of 79% (TN/(TN+FP)), a precision of 68% (TP/(TP+FP)), and an F1-Score of 60%.

## Discussion

Despite the global implementation of healthcare decentralization, there is a notable gap in research examining the factors predicting successful workforce transitions in decentralized systems. While previous studies, such as those conducted in Lesotho, have explored healthcare workers’ perspectives as frontline service providers, they have primarily focused on descriptive analyses rather than predictive modeling of transition success factors ( Birru et al., 2024). This gap underscores the need for quantitative approaches to identify key determinants of successful workforce transitions in decentralized healthcare systems. The findings of current study provide valuable insights into the factors influencing successful workforce transitions in healthcare settings, particularly within decentralized systems. Our SVM analysis revealed several key aspects worthy of detailed discussion.

### Methodological considerations and model performance

Our cross-validated accuracy of 68.5±3.8% ( Table 3) represents a conservative and realistic assessment of model performance, falling within the acceptable range for ML-based clinical prediction and management models reported in recent systematic reviews (65-85% accuracy range) ( Lee et al., 2022; Maghami et al., 2023). This performance demonstrates an adequate discriminative ability for healthcare workforce prediction applications. The application of SMOTE to address class imbalances demonstrates methodological sophistication in handling real-world healthcare data challenges. The improvement in sensitivity from 46.1% to 54.3% provides more balanced performance metrics, which is crucial for practical deployment in workforce management decisions, where identifying successful transitions is essential for resource allocation and policy planning. The stability of the feature importance rankings across the validation folds (coefficient of variation <0.15) strengthens the confidence in the identified predictors. The consistent emergence of competitive compensation and career development opportunities as top predictors aligns with established workforce retention research, demonstrating the significant positive effects of compensation and career development on employee retention rates ( Houssein et al., 2020), while providing quantitative validation through machine learning approaches.

### Model selection justification and comparative analysis

Comparative cross-validation analysis confirmed the appropriateness of the linear SVM selection over the alternative algorithms. While Random Forest showed competitive performance (65.8% accuracy), the linear SVM’s combination of superior accuracy (68.5%) and interpretable feature weights provided optimal value for healthcare administrative decision-making. The linear kernel’s performance suggests that workforce transition success can be effectively modeled through linear combinations of organizational and individual factors, supporting the interpretability of our findings. The superior performance of SVM compared to traditional logistic regression (66.2% accuracy) validates the application of machine learning approaches in healthcare workforce management. This advancement aligns with recent trends in healthcare analytics, where sophisticated algorithms increasingly outperform conventional statistical methods ( Bikku, 2020).

Comparative cross-validation analysis confirmed the appropriateness of the linear SVM selection over the alternative algorithms. While Random Forest showed competitive performance (65.8% accuracy), the linear SVM’s combination of superior accuracy (68.5%) and interpretable feature weights provided optimal value for healthcare administrative decision-making. The linear kernel’s performance suggests that workforce transition success can be effectively modeled through linear combinations of organizational and individual factors, supporting the interpretability of our findings. The superior performance of SVM compared to traditional logistic regression (66.2% accuracy) validates the application of machine learning approaches in healthcare workforce management. This advancement aligns with recent trends in healthcare analytics, where sophisticated algorithms increasingly outperform conventional statistical methods ( Bikku, 2020).

A. Primary determinants of workforce transitions
  Our cross-validated SVM analysis revealed that successful workforce transitions in healthcare decentralization are primarily driven by a combination of financial incentives and professional development opportunities. The emergence of competitive compensation (Benefits5: 0.427) as the strongest predictor, followed by career development opportunities (Welfare30: 0.358) and fair promotion processes (Benefits4: 0.336), demonstrates the dual importance of immediate financial benefits and long-term career prospects. This finding aligns with Brennan and Abimbola’s observations that health workers’ mobility in decentralized systems is significantly influenced by salary differentials, with workforce movement patterns strongly associated with compensation variations across jurisdictions ( Brennan & Abimbola, 2023).
B. Role of educational background
  The emergence of education as the only demographic variable among top predictors (feature weights: 0.236) contributes a distinct dimension to the hyperplane, suggesting that while individual characteristics influence transition success, their impact creates a smaller angular component in the overall decision boundary compared to organizational factors. This geometric interpretation provides a new perspective on the relative importance of different factor categories. Our findings showed that education level emerged as the sole influential demographic factor for personnel decentralization success, which presents an interesting pattern requiring further interpretation. This could be attributed to the sample characteristics, where bachelor’s degree holders constituted the majority (around 70%) of participants. The dominance of this educational demographic might have influenced the SVM model’s variable importance outcomes. However, it’s important to note that the current literature does not provide direct evidence explaining why education level would be uniquely influential while other demographic factors show less importance in personnel decentralization success. This finding suggests a potential area for future research to explore the specific mechanisms through which education level impacts decentralization outcomes in healthcare organizations. The predominance of bachelor’s degree holders (more than 70%) in our sample merits careful interpretation of the SVM results. As highlighted by Batuwita and Palade (2013) and in Haikal et al. (2024), SVM models can be sensitive to unbalanced predictor distributions, potentially leading to classification bias toward the majority class. This methodological consideration suggests that the apparent significance of education level as a predictor might partially reflect the dataset’s compositional characteristics rather than solely representing its intrinsic importance in personnel decentralization success. This understanding underscores the importance of considering data distribution patterns when interpreting machine learning outcomes in organizational research.

### Practical implications for workforce management

These findings offer generalizable lessons for several countries implementing healthcare decentralization, specifically in predicting and managing successful personnel transfers. The predictive modeling approach enables the proactive identification of personnel at risk of unsuccessful transitions, allowing for targeted interventions and support mechanisms. Our findings align with Thailand’s National Health Security Act of 2019, which emphasizes local autonomy in healthcare management while maintaining service quality standards. To our knowledge, this study represents the first systematic investigation using state-of-the-art machine learning techniques to predict healthcare personnel transition success in a decentralization context, moving beyond traditional descriptive analyses of workforce perspectives. By applying SVM methodology to analyze personnel transfers from central to local administration, we provide novel insights into the quantitative prediction of transition success factors, contributing to the growing body of evidence in healthcare workforce management during decentralization reforms. The identified predictors provide evidence-based guidance for designing comprehensive support packages that balance immediate financial incentives with long-term career development opportunities. This predictive modeling approach can be adapted by other healthcare systems undertaking decentralization to assess their workforce’s readiness for transition and identify specific support mechanisms needed for successful personnel transfers.

### Limitations and considerations

Several methodological limitations should be considered when interpreting our findings. First, while our cross-validation approach provides robust performance estimates, the single-country nature of our study limits its external generalizability. Future multi-country validation studies should assess the transferability of models across different healthcare systems and decentralization policies. Second, our cross-sectional design prevented the assessment of temporal causality between predictors and transition outcomes. Longitudinal studies tracking personnel across multiple transition phases would enable dynamic prediction modeling and stronger causal inference. Third, despite implementing SMOTE to address class imbalance, the moderate cross-validated sensitivity (around 54%) suggests that there is room for improvement in identifying successful transitions. Advanced ensemble methods or deep learning approaches may achieve better balanced performance, particularly given recent advances in healthcare prediction modeling ( Bikku, 2020; Bikku et al., 2025). Fourth, the predominance of bachelor’s degree holders may have influenced the apparent predictive importance of education. Future studies with stratified sampling across educational levels could clarify this relationship and reduce the potential sampling bias effects on SVM classification. Finally, our linear kernel assumption implies a linear relationship between the predictors and outcomes. Nonlinear kernel exploration or alternative ML approaches may reveal complex interaction patterns in workforce transition dynamics, potentially improving predictive accuracy beyond our current cross-validated results.

## Conclusion

This study successfully developed and validated a Support Vector Machine model for predicting healthcare workforce transition success under decentralization, achieving a moderate classification accuracy of 68.5±3.8%. The predictive modeling approach with 10-fold cross-validation effectively identified key determinants of successful transitions, with competitive compensation (0.427) and career development opportunities (0.358) emerging as the strongest predictors and most stable predictors across the validation folds. This finding highlights the critical role of both financial incentives and professional growth opportunities in facilitating successful workforce transitions.

The model revealed that organizational support mechanisms, particularly those related to compensation and career development, have greater predictive power than individual characteristics, though employee qualifications (0.236) emerged as a significant contributor to transition success. These insights provide evidence-based guidance for healthcare administrators implementing decentralization policies. The implementation of SMOTE to address class imbalance improved the sensitivity to 54.3±4.8%, providing a more balanced performance for practical workforce management applications. The cross-validation results demonstrated model stability and generalizability, with consistent feature importance rankings across all validation folds. The superior performance compared to alternative algorithms (logistic regression: 66.2%, random forest: 65.8%) validates the SVM approach for healthcare workforce prediction applications.

These findings contribute to the understanding of healthcare workforce adaptation and offer practical tools for optimizing transition processes in decentralized healthcare systems.

### Ethical approval and consent

The study protocol was approved by the Ethics Committee for Human Research of Prachomklao College of Nursing (PCKCN), Praboromarajchanok Institute, Ministry of Public Health, Thailand (approval number: REC No. 13/2566, dated March 23, 2023). All participants provided written informed consent prior to data collection. The consent process and study protocols were conducted in accordance with the Declaration of Helsinki.

### Declaration of generative AI and AI-assisted technologies in the writing process

During the preparation of this work, the author(s) used Claude 3.5 Sonnet to assist with language refinement, grammar correction, and structural organization of the manuscript. All AI-generated content was critically reviewed, verified, and edited by the authors to maintain scientific accuracy and authenticity.

## Data Availability

The datasets used during this study are not publicly available due to privacy concerns and ethical restrictions on participant data as specified by the Ethics Committee for Human Research of Prachomklao College of Nursing (PCKCN). However, the data are available from the corresponding author ( chinaks@umich.edu) upon reasonable request with approval from the PCKCN Ethics Committee, agreement to maintain participant confidentiality, and compliance with data protection protocols outlined in ethics approval (REC No. 13/2566). The questionnaire and STROBE checklist are available as Extended data on OSF: Support vector machine-based prediction model for healthcare workforce transition success under decentralization ( https://doi.org/10.17605/OSF.IO/2HF3N) ( Sujimongkol & Sarakshetrin, 2024). This project contains the following underlying data:
• Questionnaire_Thai.pdf (Original Thai version questionnaire) • Questionnaire_English.pdf (English translated questionnaire) • STROBE_checklist.pdf (STROBE checklist for cross-sectional study) Data are available under the terms of the Creative Commons Zero “No rights reserved” data waiver (CC0 1.0 Public domain dedication).
