# Supplementary material for: Support Vector Machine-Based Prediction Model for Healthcare Workforce Transition Success Under Decentralization
Source: F1000Res. 2025 Sep 24;14:49. Originally published 2025 Jan 9. [Version 2] doi: 10.12688/f1000research.160378.2 (PMC12559843; doi:10.12688/f1000research.160378.2)
Supplement: Peer review history

# Reviewer report: Reviewer response for version 2

**DOI:** 10.5256/f1000research.187886.r425329

**Published:** 2025-11-03

**Authors:** Franklin Akwasi Adjei

**Affiliations:** 1 University of Wyoming, Wyoming, USA

**1. Statistical Analysis and Methodology**

**Appropriateness of Statistical Methods**

 The statistical analysis in this study is effectively implemented, especially the use of the Support Vector Machine for predicting healthcare workforce transitions. The choice of SVM is appropriate given its strength in handling complex, multidimensional data, which is crucial for predicting workforce outcomes influenced by multiple interrelated factors (e.g., compensation, career development, education). The study uses cross-validation to assess the model’s performance, which is essential in machine learning to prevent overfitting and ensure that the results generalize well to unseen data.

 The cross-validated accuracy of 68.5% ± 3.8% is a reasonable result for a healthcare workforce prediction model and aligns with typical performance levels reported in machine learning studies in healthcare (Lee et al., 2022). However, it would be helpful to include a bit more detail about what accuracy levels are considered acceptable when healthcare personnel make transitions. This can make the discussion clearer and more comprehensive. Given the consequences of misclassifying transitions, it would be useful to emphasize further the trade-offs among accuracy, sensitivity, and specificity in healthcare applications. While the sample size (430 participants) appears sufficient, a detailed power analysis could provide greater clarity for the study. This would provide more confidence in the sample size's ability to detect significant predictors and clarify whether the study is underpowered or well-powered.

**2. Presentation and Clarity**

 The manuscript is thoughtfully organized, featuring a clear introduction, methods, results, and discussion that guide readers smoothly through the research. The structure makes it easy to follow and understand the study's progression sections. The authors provide a comprehensive overview of the study's objectives and methodology, followed by a detailed analysis of the findings. However, some sections could benefit from further clarification and elaboration. The introduction sets the context well, but it could improve by explaining the specific advantages of using SVMs for predicting workforce transitions, particularly compared to traditional statistical methods. A more explicit discussion of the limitations of traditional methods (e.g., logistic regression) and why SVM offers a better solution in this context would enhance the manuscript’s clarity.

**3. Discussion and Interpretation of Results**

 The SVM analysis strongly supports the study's findings that competitive pay, career advancement, and fair promotions are key factors in successful workforce transitions. These findings are consistent with existing research on employee retention, highlighting the importance of financial incentives and professional growth in healthcare workforce mobility. Notably, education level emerged as the sole influential demographic factor; however, the authors should be aware of possible bias due to the higher proportion of bachelor’s degree holders in the sample. This imbalance could restrict the generalizability of the results to the wider workforce. Future studies should employ stratified sampling to ensure a more representative demographic distribution.

**4. Practical Implications and Generalizability**

 The authors should discuss the potential limitations of applying the model outside of Thailand, given that decentralization processes and healthcare systems vary widely across countries. Multi-country studies would strengthen the external validity of the findings. The authors could also discuss how the model’s findings could inform broader healthcare policy decisions, such as the structuring of decentralized healthcare systems in low- and middle-income countries.

Is the work clearly and accurately presented and does it cite the current literature?

Yes

If applicable, is the statistical analysis and its interpretation appropriate?

Yes

Are all the source data underlying the results available to ensure full reproducibility?

Partly

Is the study design appropriate and is the work technically sound?

Yes

Are the conclusions drawn adequately supported by the results?

Yes

Are sufficient details of methods and analysis provided to allow replication by others?

Yes

Reviewer Expertise:

Health Research, Public Health

I confirm that I have read this submission and believe that I have an appropriate level of expertise to confirm that it is of an acceptable scientific standard, however I have significant reservations, as outlined above.

# Reviewer report: Reviewer response for version 2

**DOI:** 10.5256/f1000research.187886.r425323

**Published:** 2025-10-27

**Authors:** Ali Husnain

**Affiliations:** 1 Chicago State University, Chicago, Illinois, USA

The revised manuscript presents a well-executed and meaningful application of Support Vector Machine modeling to predict healthcare workforce transition success during Thailand’s decentralization process. The study stands out for its combination of methodological rigor and practical relevance. By using a cross-sectional design with 10-fold cross-validation and SMOTE resampling, the authors demonstrate an advanced yet transparent approach to predictive modeling in a policy context that has often relied on descriptive analyses.

 The introduction establishes a clear research gap and positions the study within current decentralization and workforce management literature. The methods section is detailed, outlining sampling strategies, variable definitions, and model comparison across linear and nonlinear kernels. The technical enhancements added in this version—particularly cross-validation, benchmarking against logistic regression and ensemble models, and the inclusion of AUC and F1-score metrics—strengthen the paper considerably.

 Results are presented with appropriate balance between quantitative reporting and interpretive discussion. The identification of compensation and professional development as key predictors is both statistically grounded and theoretically sound. The discussion effectively links findings to Thailand’s current health policy framework and comparable international experiences, giving the study broader relevance.

 There are, however, minor areas for improvement. Full reproducibility remains limited because the raw dataset cannot be openly shared; the authors could consider publishing de-identified or synthetic data to facilitate replication. A brief description of hyperparameter tuning beyond the C parameter would also enhance transparency. Finally, the discussion could be slightly condensed to reduce repetition and improve flow.

 Overall, this is a high-quality, policy-relevant study that meets publication standards. It contributes meaningful insights to the intersection of healthcare workforce research and machine learning and demonstrates thoughtful methodological execution.

Is the work clearly and accurately presented and does it cite the current literature?

Yes

If applicable, is the statistical analysis and its interpretation appropriate?

Yes

Are all the source data underlying the results available to ensure full reproducibility?

Partly

Is the study design appropriate and is the work technically sound?

Yes

Are the conclusions drawn adequately supported by the results?

Yes

Are sufficient details of methods and analysis provided to allow replication by others?

Partly

Reviewer Expertise:

Data Science, Machine Learning in Healthcare, Health Informatics, Predictive Modeling, and Public Health Systems Research.

I confirm that I have read this submission and believe that I have an appropriate level of expertise to confirm that it is of an acceptable scientific standard, however I have significant reservations, as outlined above.

# Reviewer report: Reviewer response for version 2

**DOI:** 10.5256/f1000research.187886.r417068

**Published:** 2025-10-13

**Authors:** Manaporn Chatchumni

**Affiliations:** 1 Rangsit University, Lak Hok, Pathum Thani, Thailand

I have carefully reviewed the revised version of the manuscript titled *“Support Vector Machine-Based Prediction Model for Healthcare Workforce Transition Success Under Decentralization”* by Sarakshetrin et al. The authors have satisfactorily addressed all previous comments, with significant improvements in the methodological rigor, clarity of variable definitions, model validation, and contextual discussion. The integration of national and international policy perspectives, along with enhanced technical presentation, has notably strengthened the quality and relevance of the paper.

Is the work clearly and accurately presented and does it cite the current literature?

Yes

If applicable, is the statistical analysis and its interpretation appropriate?

Partly

Are all the source data underlying the results available to ensure full reproducibility?

Yes

Is the study design appropriate and is the work technically sound?

Partly

Are the conclusions drawn adequately supported by the results?

Partly

Are sufficient details of methods and analysis provided to allow replication by others?

Partly

Reviewer Expertise:

NA

I confirm that I have read this submission and believe that I have an appropriate level of expertise to confirm that it is of an acceptable scientific standard.

# Reviewer report: Reviewer response for version 1

**DOI:** 10.5256/f1000research.176268.r393051

**Published:** 2025-08-12

**Authors:** Thulasi Bikku

**Affiliations:** 1 Amrita School of Computing Amaravati, Amrita Vishwa, Vidyapeetham, Amaravati, Andhra Pradesh, India

1. **Model Validation**: Lack of train-test splitting or k-fold cross-validation undermines reported accuracy (71.43%). Implement and report 5- or 10-fold cross-validation.
2. **SVM Development**: Unclear hyperparameter tuning (e.g., regularization parameter C) and feature weight threshold (>0.25). Provide details and justification.
3. **Sensitivity-Specificity Imbalance**: Low sensitivity (49.02%) vs. high specificity (85.37%) needs deeper analysis and strategies to improve (e.g., class weight adjustments).
4. **Sampling Bias**: Predominance of bachelor’s degree holders (71.16%) may bias education’s importance (coefficient = 0.236). Conduct stratified analysis and clarify province selection.
5. **Literature Comparison**: Limited benchmarking against other predictive models (e.g., random forests). Include comparisons and integrate international decentralization studies.
6. **Clarity**: Fix typographical errors (e.g., missing Table 3), standardize notation (e.g., ( f(x) )), and fully describe tables.
7. **Visualizations**: Add figures (e.g., feature importance plot, ROC curve) for clarity.
8. **Data Transparency**: Clarify data availability and consent process to align with open science.
9. **Limitations**: Expand discussion on cross-sectional design limitations and generalizability beyond Thailand.
10. Implement k-fold cross-validation to validate model performance.
11. Clarify hyperparameter tuning and feature weight threshold rationale.
12. Analyze low sensitivity and explore improvement strategies.
13. Address sampling bias with stratified analysis and detail province selection.
14. Benchmark against other models and integrate global decentralization studies.
15. Correct errors, standardize notation, add visualizations, and clarify data availability.
16. Add References:
  Bikku, Thulasi, and KPNV Satya Sree. "Deep learning approaches for classifying data: a review." *Journal of Engineering Science and Technology* 15.4 (2020): 2580-2594.
  Bikku, Thulasi. "Multi-layered deep learning perceptron approach for health risk prediction." *Journal of Big Data* 7.1 (2020): 50.
  BIKKU, THULASI, et al. "Healthcare Biclustering of Predictive Gene Expression Using LSTM Based Support Vector Machine." *Informing Science* 28 (2025): 12.

Is the work clearly and accurately presented and does it cite the current literature?

Yes

If applicable, is the statistical analysis and its interpretation appropriate?

Partly

Are all the source data underlying the results available to ensure full reproducibility?

Partly

Is the study design appropriate and is the work technically sound?

Partly

Are the conclusions drawn adequately supported by the results?

Partly

Are sufficient details of methods and analysis provided to allow replication by others?

Partly

Reviewer Expertise:

Bioinformatics, Deep Learning, Quantum Computing

I confirm that I have read this submission and believe that I have an appropriate level of expertise to confirm that it is of an acceptable scientific standard, however I have significant reservations, as outlined above.

## References

[1] Bikku, Thulasi, and KPNV Satya Sree. "Deep learning approaches for classifying data: a review." Journal of Engineering Science and Technology 15.4 (2020): 2580-2594.

[2] Bikku, Thulasi. "Multi-layered deep learning perceptron approach for health risk prediction." Journal of Big Data 7.1 (2020): 50. BIKKU, THULASI, et al. "Healthcare Biclustering of Predictive Gene Expression Using LSTM Based Support Vector Machine." Informing Science 28 (2025): 12.

# Response

**Published:** 2025-08-30

**Authors:** Chinakorn Sujimongkol

**Response to Reviewer 2**

**Dear Reviewer 2,**

 Thank you for your comprehensive feedback and the detailed list of recommendations. We have systematically addressed each of your concerns:

**Responses to Specific Points**

**Points 1, 10-12: Model Validation Implementation** We have completed rigorous 10-fold cross-validation analysis revealing:

- Cross-validated accuracy: 68.5±3.8%
- Optimal hyperparameter: C=1.0 (determined through grid search)
- Feature weight threshold >0.25 justified through variance stabilization analysis
- Improved sensitivity through SMOTE implementation: 54.3±4.8%

**Points 4, 13: Sampling Bias Assessment** We conducted stratified analysis by education level and detailed province selection methodology:

- Educational bias acknowledged with coefficient of variation analysis
- Province selection criteria now explicitly detailed with demographic characteristics
- Sensitivity analysis performed excluding education as predictor

**Points 5, 14: Comparative Model Analysis** Comprehensive benchmarking against alternative algorithms completed:

 We have conducted comprehensive benchmarking against alternative algorithms, including Logistic Regression, Random Forest, and Gradient Boosting, in addition to Linear SVM. The comparative results of model accuracy, sensitivity, specificity, and AUC are summarized in **Table 3 (Cross-validation performance comparison)** in the revised manuscript. In addition, the detailed classification outcomes for the optimal SVM model are presented in **Table 4 (Confusion matrix for optimal SVM model)**.

**Points 6-9, 15: Technical Corrections**

- Mathematical notation standardized: f(x) = sign(∑ᵢ₌₁ⁿ αᵢyᵢK(xᵢ,x) + b)
- Added Table 4 (confusion matrix) 
- Fixed all typographical errors
- Enhanced data availability statement

**Point 16: Literature Integration** All three suggested references have been incorporated:

- Bikku & Sree (2020): Integrated into methodology discussion on classification approaches
- Bikku (2020): Referenced in healthcare prediction context and risk assessment
- Bikku et al. (2025): Cited for advanced healthcare ML applications and comparison

**Detailed Technical Improvements**

**Cross-Validation Implementation:** The 10-fold cross-validation maintained stratified sampling to preserve class distribution across folds. Performance stability was assessed through coefficient of variation analysis, with all top predictors showing CV <0.15, indicating robust feature importance rankings.

**Class Imbalance Resolution:** SMOTE implementation during training phases generated synthetic minority class examples, improving model balance. The trade-off between sensitivity and specificity was optimized for healthcare decision-making contexts, where identifying successful transitions is crucial for workforce planning.

**Feature Stability Analysis:** Cross-validation confirmed the consistency of our top 5 predictors:

- Competitive compensation: 0.427 (CV range: 0.398-0.456)
- Career development: 0.358 (CV range: 0.334-0.382)
- Fair promotion: 0.336 (CV range: 0.312-0.360)
- Hazardous work compensation: 0.285 (CV range: 0.265-0.305)
- Educational leave: 0.252 (CV range: 0.235-0.269)

**Sincerely,**

**Chinakorn Sujimongkol**

 Corresponding author

# Reviewer report: Reviewer response for version 1

**DOI:** 10.5256/f1000research.176268.r358598

**Published:** 2025-03-28

**Authors:** Manaporn Chatchumni

**Affiliations:** 1 Rangsit University, Lak Hok, Pathum Thani, Thailand

Dear Editors,

 Thank you for the opportunity to review this manuscript. The study presents a novel and relevant application of machine learning—specifically Support Vector Machine (SVM)—to model the predictors of healthcare workforce transition success in the context of decentralization in Thailand. This topic is timely and significant, offering valuable insights into both public health workforce planning and AI-driven predictive analytics.

 While the manuscript is well-structured and contributes to a growing area of applied machine learning in health systems research, I offer the following major and minor comments to enhance its clarity, methodological rigor, and policy relevance:

 Major Comments

 1.    Model Validation and Generalizability

 The current manuscript lacks any mention of model validation (e.g., train-test split or cross-validation), which limits the credibility of the reported performance metrics (accuracy: 71.43%).

 Recommendation: Incorporate k-fold cross-validation or a separate test set to assess model generalizability and reduce potential overfitting.

 2.    Definition and Operationalization of Outcome Variable

 The manuscript references “successful transition” based on satisfaction levels, but the criteria for classification into “success” or “non-success” are not clearly defined.

 Recommendation: Provide a more explicit explanation of how the binary outcome was coded, including any threshold values or composite score criteria.

 3.    Handling of Potential Class Imbalance

 The reported sensitivity (49.02%) suggests potential imbalance in the outcome classes.

 Recommendation: Report the distribution of successful vs. unsuccessful cases and consider using resampling or weighting methods (e.g., SMOTE, class weights) to improve sensitivity.

 4.    Interpretability of the SVM Model

 While linear SVM was chosen, its interpretability compared to other models (e.g., logistic regression or decision trees) could be discussed more thoroughly.

 Recommendation: Justify the use of a linear kernel over other interpretable models and elaborate on the reliability of the identified feature weights as indicators of predictor importance.

 5.    Strengthening Contextual Discussion

 The findings could be better connected to Thailand’s decentralization reform context.

 Recommendation: Expand the discussion on how these predictors align with current national health workforce policies and potential implications for policy adaptation or intervention design.

 Minor Comments

 •    Abstract: Consider reporting key model performance metrics in the abstract for transparency.

 •    Introduction: A brief rationale for selecting SVM over other machine learning models would strengthen the justification.

 •    Tables/Figures: A confusion matrix or ROC curve figure would enhance understanding of model performance.

 •    References: Where possible, provide additional evidence to support the unique influence of demographic predictors like education.

 Overall Assessment

 This manuscript is a valuable contribution to the intersection of AI and health systems research. It is particularly relevant for policymakers and administrators navigating workforce transitions under decentralization. With the suggested revisions, the paper will meet a higher standard of methodological transparency and policy relevance.

 Sincerely,

Is the work clearly and accurately presented and does it cite the current literature?

Yes

If applicable, is the statistical analysis and its interpretation appropriate?

Partly

Are all the source data underlying the results available to ensure full reproducibility?

Yes

Is the study design appropriate and is the work technically sound?

Partly

Are the conclusions drawn adequately supported by the results?

Partly

Are sufficient details of methods and analysis provided to allow replication by others?

Partly

Reviewer Expertise:

This manuscript is a valuable contribution to the intersection of AI and health systems research. It is particularly relevant for policymakers and administrators navigating workforce transitions under decentralization. With the suggested revisions, the paper will meet a higher standard of methodological transparency and policy relevance.

I confirm that I have read this submission and believe that I have an appropriate level of expertise to confirm that it is of an acceptable scientific standard, however I have significant reservations, as outlined above.

# Response

**Published:** 2025-08-30

**Authors:** Chinakorn Sujimongkol

**Response to Reviewers**

**Response to Reviewer 1**

**Dear Reviewer 1,**

 We sincerely appreciate your thorough review and constructive feedback. Your comments have significantly improved the quality of our manuscript. Below are our detailed responses to each of your major and minor comments:

**Major Comments**

**Comment 1: Model Validation and Generalizability** *Response:* We have now implemented 10-fold cross-validation to assess model performance more rigorously. The cross-validation results show:

- Cross-validated accuracy: 68.5% (±3.8%)
- Cross-validated sensitivity: 46.1% (±5.2%)
- Cross-validated specificity: 82.4% (±4.1%)
- Area Under Curve (AUC): 0.642 (±0.045)

These metrics provide a more realistic and robust estimate of model performance and demonstrate good generalizability across different data partitions.

**Comment 2: Definition and Operationalization of Outcome Variable** *Response:* We have clarified the binary outcome definition in the Methods section. The "successful transition" was operationalized using a composite satisfaction score approach:

- Successful transition: Mean satisfaction score ≥ 3.5 across benefits and welfare domains
- Unsuccessful transition: Mean satisfaction score < 3.5
- Final distribution: Successful transitions (n=195, 45.3%), Unsuccessful transitions (n=235, 54.7%)

This threshold was determined through ROC analysis to optimize the sensitivity-specificity balance for practical application.

**Comment 3: Handling of Potential Class Imbalance** *Response:* Our analysis revealed moderate class imbalance (45.3% vs 54.7%). We implemented Synthetic Minority Oversampling Technique (SMOTE) to address this issue, resulting in improved performance:

- Improved sensitivity: 54.3% (±4.8%)
- Maintained specificity: 78.9% (±4.5%)
- Overall accuracy: 66.8% (±4.2%)
- Enhanced AUC: 0.666 (±0.042)

**Comment 4: Interpretability of the SVM Model** *Response:* We conducted comparative analysis with other interpretable models to justify SVM selection:

- Logistic Regression: 66.2% (±4.0%) accuracy
- Decision Trees: 63.1% (±4.8%) accuracy
- Random Forest: 65.8% (±4.5%) accuracy
- Gradient Boosting: 65.1% (±4.3%) accuracy

The linear SVM's superior cross-validated performance (68.5%) combined with interpretable feature weights justifies its selection over alternative approaches.

**Comment 5: Strengthening Contextual Discussion** *Response:* We have substantially expanded the discussion to include:

- Alignment with Thailand's 2019 National Health Security Act
- Connection to Ministry of Public Health's 2023-2027 Strategic Plan
- International comparisons with Brazil, Kenya, and Indonesia decentralization experiences
- Specific policy recommendations based on identified predictors

**Minor Comments**

 All minor comments have been addressed:

- Abstract now includes cross-validated performance metrics
- Introduction includes comprehensive SVM selection rationale
- Added Table 4 (confusion matrix) and Figure 1 (ROC curve analysis)
- Strengthened demographic predictor evidence with additional international citations

**Response to Reviewer 2**

**Dear Reviewer 2,**

 Thank you for your comprehensive feedback and the detailed list of recommendations. We have systematically addressed each of your concerns:

**Responses to Specific Points**

**Points 1, 10-12: Model Validation Implementation** We have completed rigorous 10-fold cross-validation analysis revealing:

- Cross-validated accuracy: 68.5±3.8%
- Optimal hyperparameter: C=1.0 (determined through grid search)
- Feature weight threshold >0.25 justified through variance stabilization analysis
- Improved sensitivity through SMOTE implementation: 54.3±4.8%

**Points 4, 13: Sampling Bias Assessment** We conducted stratified analysis by education level and detailed province selection methodology:

- Educational bias acknowledged with coefficient of variation analysis
- Province selection criteria now explicitly detailed with demographic characteristics
- Sensitivity analysis performed excluding education as predictor

**Points 5, 14: Comparative Model Analysis** Comprehensive benchmarking against alternative algorithms completed:

**Model**

**Accuracy (%)**

**Sensitivity (%)**

**Specificity (%)**

**AUC**

 Linear SVM

 68.5 ± 3.8

 46.1 ± 5.2

 82.4 ± 4.1

 0.642

 Logistic Regression

 66.2 ± 4.0

 43.8 ± 5.0

 81.1 ± 4.3

 0.625

 Random Forest

 65.8 ± 4.5

 48.9 ± 5.8

 77.2 ± 4.8

 0.631

 Gradient Boosting

 65.1 ± 4.3

 47.2 ± 5.5

 78.5 ± 4.2

 0.628

**Points 6-9, 15: Technical Corrections**

- Mathematical notation standardized: f(x) = sign(∑ᵢ₌₁ⁿ αᵢyᵢK(xᵢ,x) + b)
- Added Table 4 (confusion matrix) and Figure 2 (feature importance plot)
- Fixed all typographical errors
- Enhanced data availability statement

**Point 16: Literature Integration** All three suggested references have been incorporated:

- Bikku & Sree (2020): Integrated into methodology discussion on classification approaches
- Bikku (2020): Referenced in healthcare prediction context and risk assessment
- Bikku et al. (2025): Cited for advanced healthcare ML applications and comparison

**Detailed Technical Improvements**

**Cross-Validation Implementation:** The 10-fold cross-validation maintained stratified sampling to preserve class distribution across folds. Performance stability was assessed through coefficient of variation analysis, with all top predictors showing CV <0.15, indicating robust feature importance rankings.

**Class Imbalance Resolution:** SMOTE implementation during training phases generated synthetic minority class examples, improving model balance. The trade-off between sensitivity and specificity was optimized for healthcare decision-making contexts, where identifying successful transitions is crucial for workforce planning.

**Feature Stability Analysis:** Cross-validation confirmed the consistency of our top 5 predictors:

- Competitive compensation: 0.427 (CV range: 0.398-0.456)
- Career development: 0.358 (CV range: 0.334-0.382)
- Fair promotion: 0.336 (CV range: 0.312-0.360)
- Hazardous work compensation: 0.285 (CV range: 0.265-0.305)
- Educational leave: 0.252 (CV range: 0.235-0.269)

**Summary of Manuscript Revisions**

**New Sections Added:**

1. **Cross-Validation Methodology** (Methods section)
2. **Comparative Model Analysis** (Results section)
3. **Enhanced Policy Context** (Discussion section)
4. **Comprehensive Limitations** (Discussion section)

**New Tables and Figures:**

- **Table 3:** Cross-validation performance comparison
- **Table 4:** Confusion matrix for optimal SVM model
- **Figure 1:** ROC curve analysis with AUC comparisons
- **Figure 2:** Feature importance visualization with confidence intervals

**Enhanced Content:**

- **Methods:** Added 756 words covering validation procedures
- **Results:** Added 445 words with cross-validation findings
- **Discussion:** Added 623 words with policy context and international comparisons
- **References:** Added 8 new citations including all suggested sources

**Key Methodological Improvements:**

1. Rigorous 10-fold cross-validation implementation
2. Class imbalance handling through SMOTE
3. Hyperparameter optimization documentation
4. Comparative model benchmarking
5. Feature stability assessment
6. Enhanced interpretability analysis

**Policy Relevance Enhancement:**

1. Direct alignment with Thailand's health policy framework
2. International contextualization with comparable systems
3. Evidence-based recommendations for implementation
4. Quantitative guidance for resource allocation

**Technical Quality Improvements:**

- Standardized mathematical notation
- Corrected typographical errors
- Enhanced data transparency
- Improved statistical reporting standards

We believe these comprehensive revisions have significantly strengthened the manuscript's methodological rigor while maintaining its practical relevance for healthcare workforce management in decentralization contexts. The study now provides robust evidence for evidence-based policy development and implementation.

**Sincerely,**

 Chinakorn Sujimongkol
